# An experimental intraradicular biofilm model in the pig for evaluating irrigation techniques

**DOI:** 10.1186/s12903-021-01536-w

**Published:** 2021-04-07

**Authors:** Toshinori Tanaka, Yoshio Yahata, Keisuke Handa, Suresh V. Venkataiah, Mary M. Njuguna, Masafumi Kanehira, Tatsuya Hasegawa, Yuichiro Noiri, Masahiro Saito

**Affiliations:** 1 Division of Operative Dentistry, Department of Ecological Dentistry, Tohoku University Graduate School of Dentistry, 4-1 Seiryo-machi, Aobaku, Sendai, Miyagi 980-8575 Japan; 2 Division of Molecular Biology and Oral Biochemistry, Department of Oral Science, Graduate School of Dentistry, Kanagawa Dental University, 82 Inaoka-cho, Yokosuka, Kanagawa 238-8580 Japan; 3 Division of Cariology, Operative Dentistry and Endodontics, Department of Oral Health Science, Niigata University Graduate School of Medical and Dental Sciences, 2-5274 Gakko-cho-dori, Chuo-ku, Niigata, Niigata 951-8514 Japan

**Keywords:** Debridement, Intraradicular biofilm model, Irrigant activation technique, Laser-activated irrigation, Pig model, Root canal disinfection

## Abstract

**Background:**

We established an in vivo intraradicular biofilm model of apical periodontitis in pigs in which we compared the efficacy of different irrigant activation techniques for biofilm removal.

**Methods:**

Twenty roots from the deciduous mandibular second premolar of 5 male pigs were used. After pulpectomy, canals were left open for 2 weeks and then sealed for 4 weeks to enable the development of an intracanal biofilm. The intraradicular biofilms was evaluated using SEM and bacterial 16S rRNA gene-sequencing. To investigate the efficacy of biofilm removal, root canal irrigations were performed using conventional needle, passive ultrasonic, subsonic, or laser-activated irrigation. Real-time PCR was conducted to quantitate the remaining biofilm components. Statistical analysis was performed using ANOVA followed by a *Tukey kramer* post-hoc test with α = 0.05.

**Results:**

The pulp exposure model was effective in inducing apical periodontitis and SEM analysis revealed a multi-layer biofilm formation inside the root canal. 16S rRNA sequence analysis identified Firmicutes, Bacteroidetes, and Fusobacteria as the predominant bacterial phyla components, which is similar to the microbiome profile seen in humans. None of the tested irrigation techniques completely eradicated the biofilm components from the root canal, but the subsonic and laser-activated irrigation methods produced the lowest bacterial counts (*p* < 0.05).

**Conclusions:**

An experimental intraradicular biofilm model has been successfully established in pigs. Within the limitations of the study, subsonic or laser-activated irrigation demonstrated the best biofilm removal results in the pig system.

## Background

Infection and biofilm formation in the root canal system are important causative factors for apical periodontitis. Current treatments aim to eliminate or substantially decrease the bacterial load within this system [1, 2]. Current mechanical instrumentation technology has produced an insufficient reduction in bacteria due to the complexity of the root canal system [3–5]. Hence, the actions of irrigating solutions are required to reduce the bacterial load to a subcritical level that will promote wound healing of the periapical tissue.

Sodium hypochlorite (NaOCl) is the most commonly used root canal irrigant that has disinfecting capacity and the ability to disrupt biofilms and dissolve organic tissues [5–7]. Conventional needle irrigation (CNI) is the standard procedure for the delivery of an NaOCl solution but this method can entrap air bubbles through a vapor lock effect [8, 9], or create an unexchanged irrigant area which becomes a “dead water” zone [10]. Moreover, delivering irrigants close to the root canal apex can cause severe pain and acute inflammation. Extruding irrigants such as NaOCl through the extraradicular area can also sometimes lead to hospitalization due to the high toxicity of this compound towards vital tissues [11]. The establishment of an alternative irrigation technique that can efficiently remove the infection source in the root canal system is thus essential for improving the efficacy and outcomes of endodontic treatments.

Various irrigant activation techniques have been proposed to improve irrigant distribution through the canal system, and enhance their antibacterial and antibiofilm capacity [12]. Ultrasonics, sonics and lasers are widely accepted methods of activating irrigants by applying an external mechanical force. Ultrasonically activated irrigation (UAI) utilizes small noncutting files that oscillate freely in the shaped canals via ultrasonic frequencies (25–30 kHz) that activate irrigants through acoustic streaming [13]. Sonic irrigation also produces a hydrodynamic phenomenon through oscillating movements at frequencies of 1–10 kHz [12]. Although these techniques are more effective than CNI, the delivery and activation of irrigants through the entire root canal system remains challenging. Laser-activated irrigation (LAI) using an Er:YAG laser has been introduced as an alternative modality for activated root canal irrigation, which can uniquely produce transient cavitation in the liquid through the optical breakdown caused by strong absorption of the laser energy [14, 15]. Hence, a pulsed Er:YAG laser evokes significant fluid movement inside the canal causing shock waves in the solution at the point of collapse, and the subsequent induction of acoustic streaming as a secondary cavitation [16–18]. LAI has been shown to be more effective in artificial biofilm reduction than either CNI or UAI [14, 19–22]. In contrast, Christo et al. found no significant differences in the ability of CNI and LAI to disinfect artificial biofilms [23], indicating that the optimal biofilm removal technique is still a point of contention.

The destruction of biofilms is a crucial requirement for reducing the microbial load in the root canal system. The microbiota of an infected root canal is typically polymicrobial and bacteria in a mature microbiota do not exist as separate colonies or in planktonic form, but as integrated communities attached to the root canal walls as biofilms [24, 25]. These attached biofilms are embedded within a self-produced extracellular polymeric matrix which is resistant to root canal irrigants [26, 27]. Numerous studies have been conducted to evaluate the efficacy of different irrigation protocols using an ex vivo extracted tooth, an in vitro plastic tooth model or computational fluid dynamics [28–32]. An artificial biofilm model developed using *Enterococcus faecalis*, which is a very relevant species for recurrent periapical pathosis, has also been developed to evaluate the efficacy of root canal irrigation systems [6, 33]. However, these ex vivo and in vitro studies have mainly involved monospecies biofilms and were therefore limited in terms of providing insights into removing biofilms that actually form in infected root canal systems.

Mouse and rat experimental models have also been developed to induce biofilm formation inside the root canal system by opening a pulp chamber [34]. These systems have provided a good understanding of the expansion and inflammation processes and pathways leading to a periapical lesion. Notably however, rodent teeth are too small to test the effectiveness of root canal irrigation in humans. In contrast, the pig is a useful animal model to evaluate the biofilm removal efficiency of root canal irrigation techniques because of the similar physiologic characteristics and tooth morphologies between pigs and humans [35]. By focusing on in vivo system with irrigation only, we aimed in our current study to develop an intraradicular biofilm and apical periodontitis model in the pig and use this system to compare the efficacy of biofilm removal between different irrigant activation techniques that can be applied in humans.

## Methods

### Ethics statement

This study was reviewed and approved by the Animal Care and Use Committees of Tohoku University Graduate school of Dentistry (Permit No. 2017 DnA-024). All animal experiments and procedures were conducted in accordance with the Regulations for Animal Experiments and Related Activities at Tohoku University. In our animal facility, the light is turned on at 8.00am and turned off at 6.00 pm. Water was available to the pigs ad libitum and a normal diet (Grandeal B; Zennoh Feed Mills of the Tohoku District, Miyagi, Japan) was provided 3 times daily. All dental interventions for each experimental tooth were performed under sodium pentobarbital anesthesia (10 mg/kg, IV) followed by inhaled sevoflurane (2–5%) with local injections of 2% lidocaine (1.8 ml, SC) to minimize pain.

### Induction of periapical bone defects in the experimental pigs

**Fig. 1:**
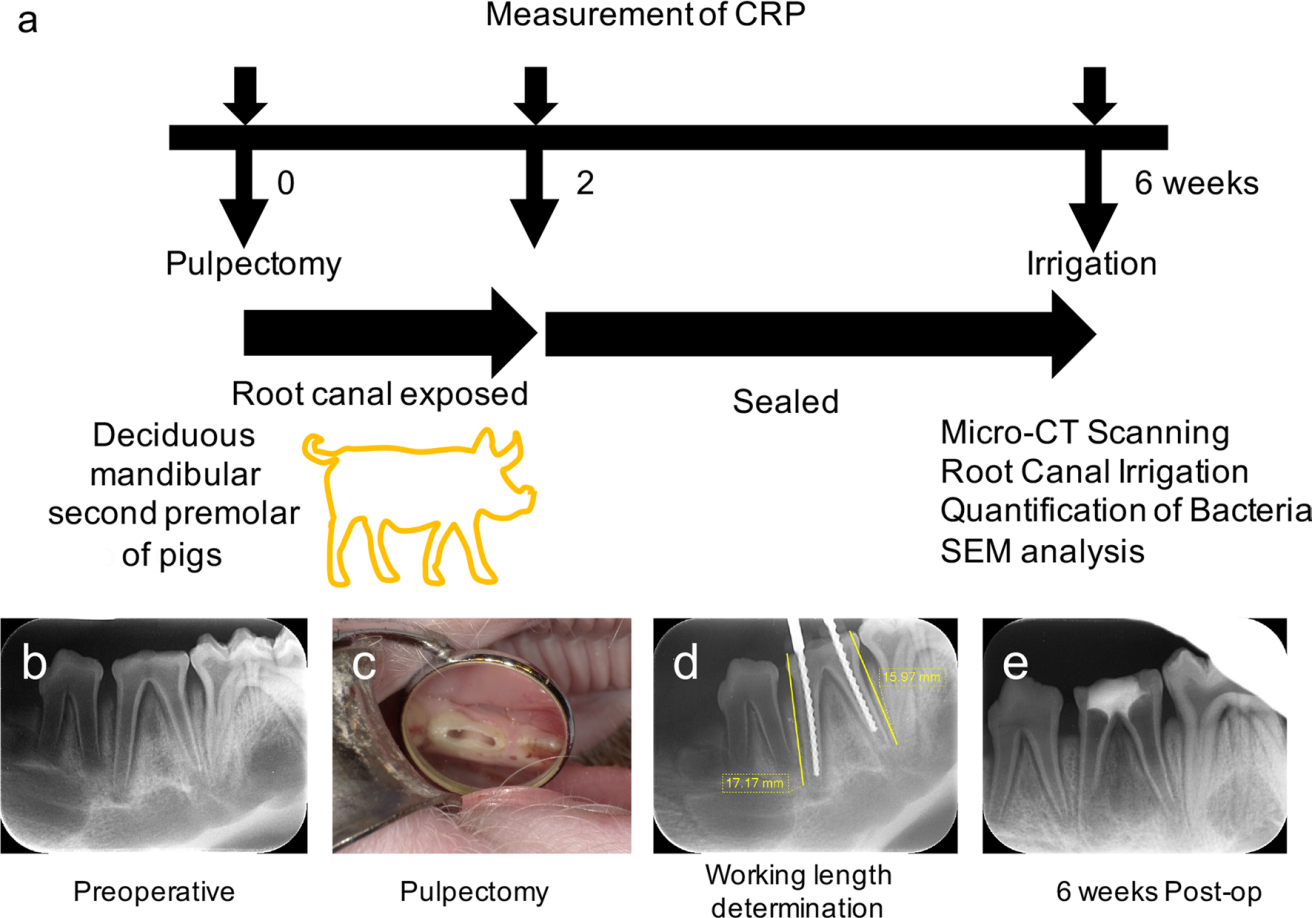
Experimental procedures for generating the intraradicular biofilm pig model. **a** CRP measurements were made at 0, 2, and 6 weeks after the pulpectomy to evaluate the inflammatory conditions in the pigs. Root canal irrigation was performed at 6 weeks. Micro-CT scanning, quantification of bacteria, and SEM analysis were performed to evaluate the periapical bone defect and intracanal infection. **b** A periapical radiograph (PA) taken prior to the pulpectomy. **c** The access cavity was exposed to enable bacterial contamination from the oral environment. **d** Working lengths were determined from the PAs. **e** The access cavity was sealed with cement and composite resin at 2 weeks and maintained for a further 4 weeks. CRP: C-reactive protein assay

The experimental protocols used in our current investigations are outlined in Fig. 1 and Table 1. Five two-month-old male pigs (Large white ☓ Landrace breed cross) were obtained from Japan SLC Inc. (Shizuoka, Japan). Twenty roots from 10 lower deciduous mandibular second premolars were used in the experiments. All procedures were performed using surgical loupes with LED light (EyeMag PRO; Carl Zeiss, Jena, Germany). Briefly, after induction of anesthesia, the occlusal surfaces were flattened with a straight bur and electric engine (Ti-Max X95; NSK, Tochigi, Japan) to prevent tooth fracture and for ease of working length determination. Following access cavity preparation and straight-line access with burs, a pulpectomy was performed with 6% sodium hypochlorite and K files. Pulp tissue was then removed, and the working length was determined with a radiograph (Fig. 1d). After subsequent chemo-mechanical debridement, canals were exposed to the oral environment for 2 weeks, after which coronal openings were sealed with hydraulic temporary filling material (Lumicon; Kulzer Japan, Tokyo, Japan) and composite resin (MI Flow II; GC, Tokyo, Japan) to create an anaerobic intracanal environment for 4 weeks. At 6 weeks after the pulpectomy, the experiments were performed, and the pigs were sacrificed. The induced periapical bone defects were scanned with a micro-CT device (ScanXmate E090; Comscantecno, Kanagawa, Japan). Six roots of the periapical bone defects were scanned, and the defect volumes were analyzed and quantified using image analysis software (TRI/3D-BON; Ratoc System Engineering, Tokyo, Japan). The lesion area was defined by configuring the boundary radiopaque area including the bone and teeth that surrounded the lesion. The lesion volume was then calculated as the region enclosed by the bounded radiopaque area. During the experimental process, blood samples were taken from 3 pigs prior to the pulpectomy, and at 2 and 6 weeks after this procedure, to determine the inflammation stage via a C-Reactive Protein Assay (CRP).

**Table 1:** Group distribution and experimental analysis

	microCT	CRP	Experimental groups									SEM	qPCR	16S rRNA
			Positive control	CNI		EA		UAI		LAI				
Pig 1	+	+	n = 4									n = 1	n = 3	n = 1
Pig 2	+	+		n = 4								n = 1	n = 3	
Pig 3	+	+				n = 4						n = 1	n = 3	
Pig 4								n = 4				n = 1	n = 3	
Pig 5										n = 4		n = 1	n = 3	

Twenty roots from 10 lower deciduous mandibular second premolars were analyzed from 5 pigs. Periapical bone defects and the inflammation stage were confirmed by micro CT and CRP in 3 animals. For irrigation comparisons, 4 canals (n = 4) were obtained from the lower deciduous mandibular second premolar of each pig. One distal root from each group was observed by SEM. The remaining three roots were used for qPCR. 16S rRNA was analyzed from one root of the positive control group. CRP: C-reactive protein assay; CNI: Conventional needle irrigation; EA: EndoActivator; UAI: Ultrasonically activated irrigation; LAI: Laser-activated irrigation

### Irrigation protocol

At 6 weeks after the pulpectomy procedure, the pigs were anesthetized to undergo root canal irrigation. For the positive control group (n = 4), teeth were extracted and intracanal biofilms were evaluated by SEM observations (n = 1) and real-time PCR (n = 3). In the other groups, the dental calculus was removed prior to treatment using an ultrasonic tip and device (Solfy F; Morita, Kyoto, Japan), and teeth were isolated with a rubber dam clamp (#212; Hu-friedy, Chicago, IL, USA) and rubber dam sheet (KSK Dental Dam Medium; Dentech, Tokyo, Japan). Aseptic conditions were established by cleaning the tooth with 3% hydrogen peroxide and 6% sodium hypochlorite, as described by Ng et al. [36] with some modifications. After temporary filling material removal, the teeth were randomly divided into 4 groups for root canal irrigation as follows: conventional 30 gauge close-ended needle irrigation (ProRinse Endo Irrigation Needles; Dentsply Sirona, PA, USA) with 6% sodium hypochlorite (CNI), CNI + ultrasonic activation (Solfy F; Morita) using a #15/02 tip (UAI), CNI + sonic activation (EndoActivator, Dentsply Sirona) with a #15/02 tip (EA), and CNI + laser activation (LAI) with a 2.94 µm wavelength Er:YAG laser (Erwin AdvErL EVO; Morita). The randomization sequence was created using a computer-generated list using Excel 2016 (Microsoft, Redmond, WA, USA). Mechanical instrumentation was not performed during the root canal irrigation.

In the CNI group (n = 4), canals were irrigated for 30 s (1 mL) and then left for 30 s, which is a procedure modified from the protocol of Al-Jadaa et al. [31]. This cycle was repeated 5 times (total procedure time, 5 min). In the UAI group (n = 4), canals were irrigated for 30 s (1 mL) with a 30G close-ended needle and irrigants were activated for 30 s ultrasonically (ENDO mode; power 10 [ca. 29.5 kHz]). This cycle was repeated 5 times (total procedure time, 5 min). The EA and LAI methods (n = 4 each) involved the same procedures as UAI but the irrigants were instead activated with EndoActivator (power; high [ca. 10 kHz]) and using an Er:YAG laser, respectively. For LAI, the activation was operated at a 50 mJ pulse energy, 20 Hz frequency, and 300 µsec pulse rate. The optic fiber (R300T, Morita) was 14 mm in length from the handpiece with a 300 μm fiber diameter and had a conical tip at the approximately 160 μm from the tip with an 84 degree angle. The tips of irrigation needles, ultrasonic device, EndoActivator, and Er:YAG laser were all placed 3 mm short from the working length and gently moved back and forth during irrigation and activation.

Following irrigation, each root canal was rinsed with 2 mL saline for 30 s. The access cavities were then sealed again with composite resin and the pigs were euthanized with a lethal dose of potassium chloride (0.25 mEq/kg, IV) under deep general anesthesia for tooth extraction. Prior to tooth extraction, we performed calculous removal and tooth cleaning to reduce the risk of bacterial contamination. The crowns were removed with a disc bur, and any remaining bacterial infection in each root was evaluated by SEM (n = 1 each) and by real-time PCR (n = 3 each) to determine the bacterial count.

### Scanning electron microscopy

The remaining debris and biofilm on the canal wall were evaluated by SEM. The crown of each experimental tooth was cut and removed using a diamond disc to separately obtain the mesial and distal roots. One distal root from each experimental sample was grooved longitudinally on the outer surface with a diamond disc and then split into two halves with a chisel. The samples were then prepared for SEM observations according to a previously described method [37, 38]. Briefly, the samples were fixed with 2.5% glutaraldehyde for more than 24 h, rinsed with PBS three times, and then treated with 1-ethyl-3-methyl-imidazolumtetrafluoroborate. After absorption of the excess, samples were dried in a vacuum desiccator for 1 day and slightly sputter-coated with platinum. The surfaces of each sample were inspected using a VE-8800 scanning electron microscope (Keyence Inc., Osaka, Japan) at a 10 kV acceleration, and the images were obtained at 30×, 1000×, and 5000× magnifications.

### DNA extraction

DNA extractions from positive control and test group samples were performed using the remaining roots. Briefly, the bacteria from the outer root surface were removed using a dental curette (YDM, Tokyo, Japan). The roots were then frozen in liquid nitrogen and crushed to powder using an SK mill (Tokken, Chiba, Japan). Total DNA was extracted from each powdered root sample using a Cica Genesus DNA extraction Kit (Kanto Chemical Co.; Tokyo, Japan) in accordance with the manufacturer’s instructions.

### Bacterial 16S rRNA gene analysis

The profile of the microbiota in the intraradicular biofilm from the positive control was confirmed by 16S rRNA gene analysis. Bacterial sequencing analysis was conducted as described by Reyes et al. [33]. Briefly, the V3-V4 region of 16S rRNA was amplified using 16S (V3-V4) metagenomic library construction kit for NGS (Takara Bio Inc, Shiga, Japan) using the following primer pair: 341F (5′-TCGTCGGCAGCGTCAGATGTGTATAAGAGACAGCCTACGGGNGGCWGCAG-3′) and 806R (5′-GTCTCGTGGGCTCGGAGATGTGTATAAGAGACAGGGACTACHVGGGTWTCTAAT-3′). Purification and quantification of the PCR amplicons were performed using Agencourt AMpure magnetic beads (Beckman Coulter, Indianapolis, IN, USA) for subsequent pyrosequencing. Index PCR assays were performed using a Nextera XT index kit (Illumina, San Diego, CA, USA) and the amplicons were again purified with the AMpure magnetic beads. An Illumine Miseq platform (Illumina) was next used to generate 250-bp paired-end sequences which were processed via the QIIME bioinformatic pipeline. After removing low-quality sequences, noise, pyrosequencing errors, and chimeras, the reads were clustered into operational taxonomic units (OTUs) with a 0.97 clustering threshold using the CD-HIT-OTU. To acquire the taxonomic classification for each OTU, representative sequences were aligned to the GreenGens database (gg_13_8) and assigned to this repository using RDP classifier v.2.2. Likewise, a homology search was performed for these sequences for assignment to the DDBJ 16S ribosomal RNA database.

### Quantification of bacterial populations in the root canal

Quantifications of the bacteria present in the root canals were performed based on previously described methods [34, 39] using the remaining powdered roots from each experimental sample. A negative control was also taken from the 2 lower deciduous mandibular first premolars without access opening. Sample powder for the negative control was produced as previously described. The presence of bacteria was verified in the experimental samples by qPCR using the bacterial primers 357F and 908R22. These assays were performed using a real-time PCR apparatus (CFX Connect; Bio-Rad Laboratories, Hercules, CA, USA). Amplifications were conducted for 40 cycles at 95 °C for 15 s followed by 65 °C for 1 min, with the fluorescence signals measured at the end of each cycle. A standard curve was generated by subjecting tenfold dilutions of a known concentration of *E. faecalis* DNA to the same qPCR protocol. The bacterial counts in all of the experimental groups were calculated using threshold cycle (Ct) values plotted against the standard curve. Statistical analysis was performed using ANOVA, followed by a *Tukey Kramer* post-hoc test using IBM SPSS version 22 (IBM SPSS, Chicago, IL, USA), with an α value of 0.05, to detect significant differences in the bacterial populations.

## Results

### Periapical lesion formation in a pig model of intraradicular biofilm formation

**Fig. 2:**
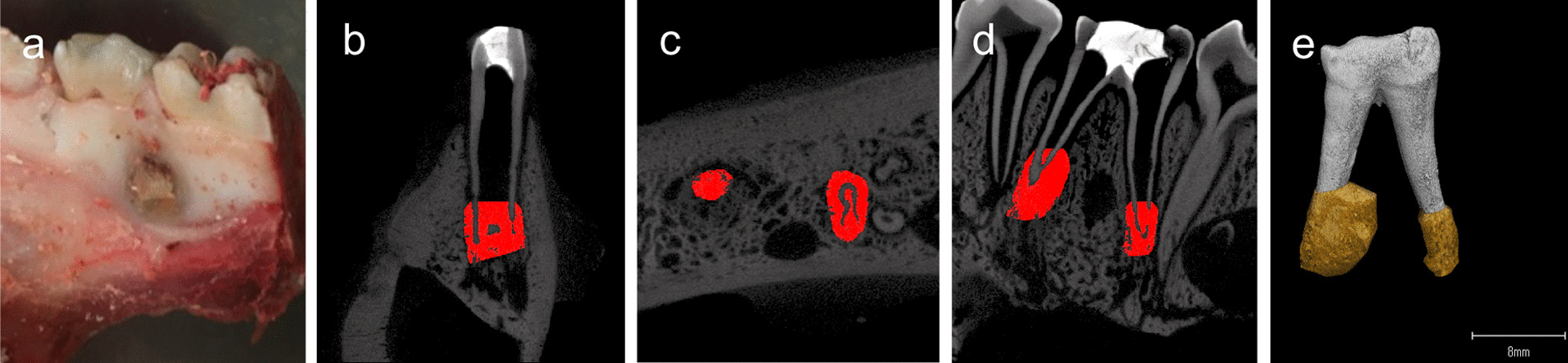
Analysis of the periapical lesions in the pig model. **a** Bone defect observed in a dissected mandibular jaw in the intraradicular biofilm pig model. **b** Frontal, **c** horizontal, and **d** sagittal views using micro CT scans and **e** three-dimensional reconstruction of a treated tooth before extraction

**Fig. 3:**
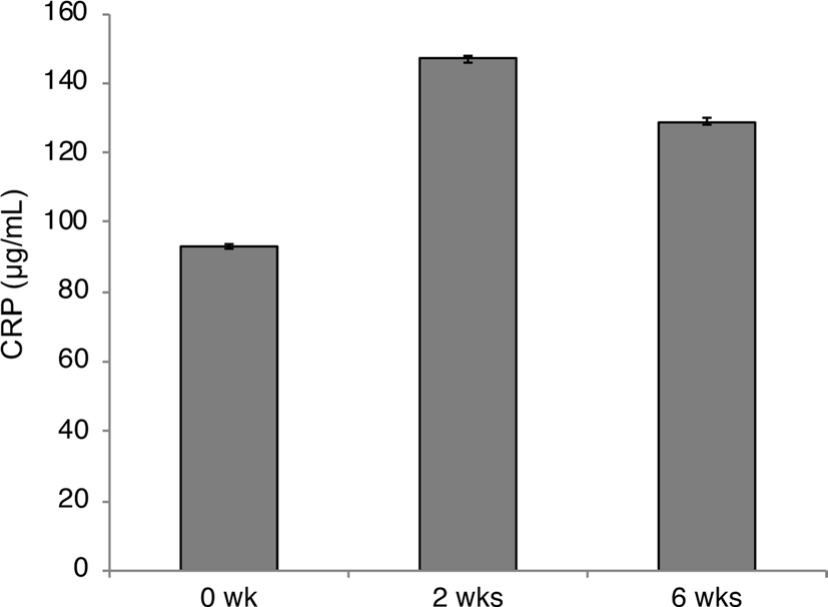
CRP measurements during periapical lesion formation. CRP measurements made at 0, 2, and 6 weeks after the pulpectomy in the pig model are shown in the graph. Data represent the means of three sample measurements. Error bars indicate standard deviations. CRP: C-reactive protein assay

Stereomicroscopic views of the dissected mandibular jaws from our intraradicular biofilm model in pigs demonstrated bone defects at the buccal side of the apex (Fig. 2a). Micro-CT analysis in frontal (Fig. 2b), horizontal (Fig. 2c), sagittal (Fig. 2d), and 3D views (Fig. 2e) revealed periapical lesion formation at both the mesial and distal roots. The mean volume of these periapical bone defects at 6 weeks after pulpectomy was 126.3 ± 97.3mm^3^. The mean CRP level was 93 μg/mL prior to the pulpectomy, 147 μg/mL at 2 weeks, and 129 μg/mL at 6 weeks (Fig. 3). The CRP level was not increased significantly but was higher at 2 weeks and lower at 6 weeks, although still above the 0 week baseline, indicating that inflammation had been induced by the periapical lesion. As an increasing CRP level was found in the first three pigs, the blood test was not conducted in subsequent pigs from the viewpoint of animal welfare.

### Characterization of the intraradicular biofilm in the pig model

**Fig. 4:**
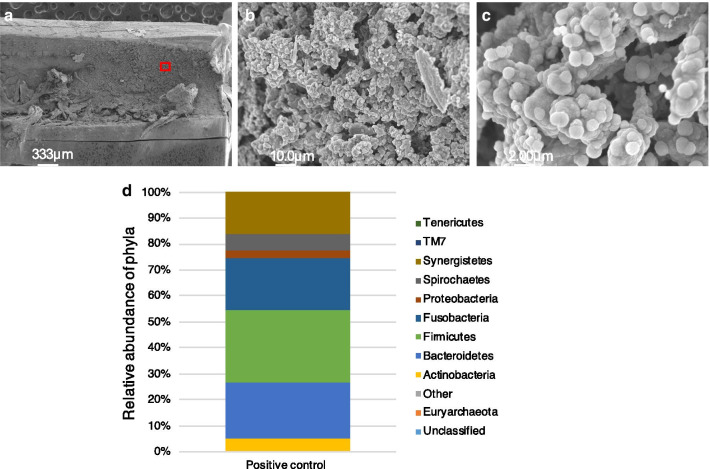
Morphological observations and bacterial 16S rRNA sequence analysis of the intraradicular biofilms in the pig model. SEM image of a root canal wall (30×) at the apical one third (**a**) and magnified views (1,000× and 5000×) (**b**, **c**) of the boxed area are shown. **d** Relative abundance of bacterial phyla. Each color on the bar indicates a different phylum

Using SEM observations of the positive control, we found that the root canal wall in our pig model was almost completely covered with debris, extracellular-matrix-like structures, and typical three-dimensional biofilm structures (Fig. 4a). Numerous cocci and some rods were also aggregated in most parts of the root canal wall area (Fig. 4b, c). Bacterial 16S rRNA sequence analysis of the biofilm formations in the root canals of the pigs identified Firmicutes (28.04%), Bacteroidetes (21.69%), and Fusobacteria (19.97%) as the major bacterial phyla, which was significant as these are also predominant in human periapical lesions (Fig. 4d).

### Effects of root canal irrigation techniques in the pig model

**Fig. 5:**
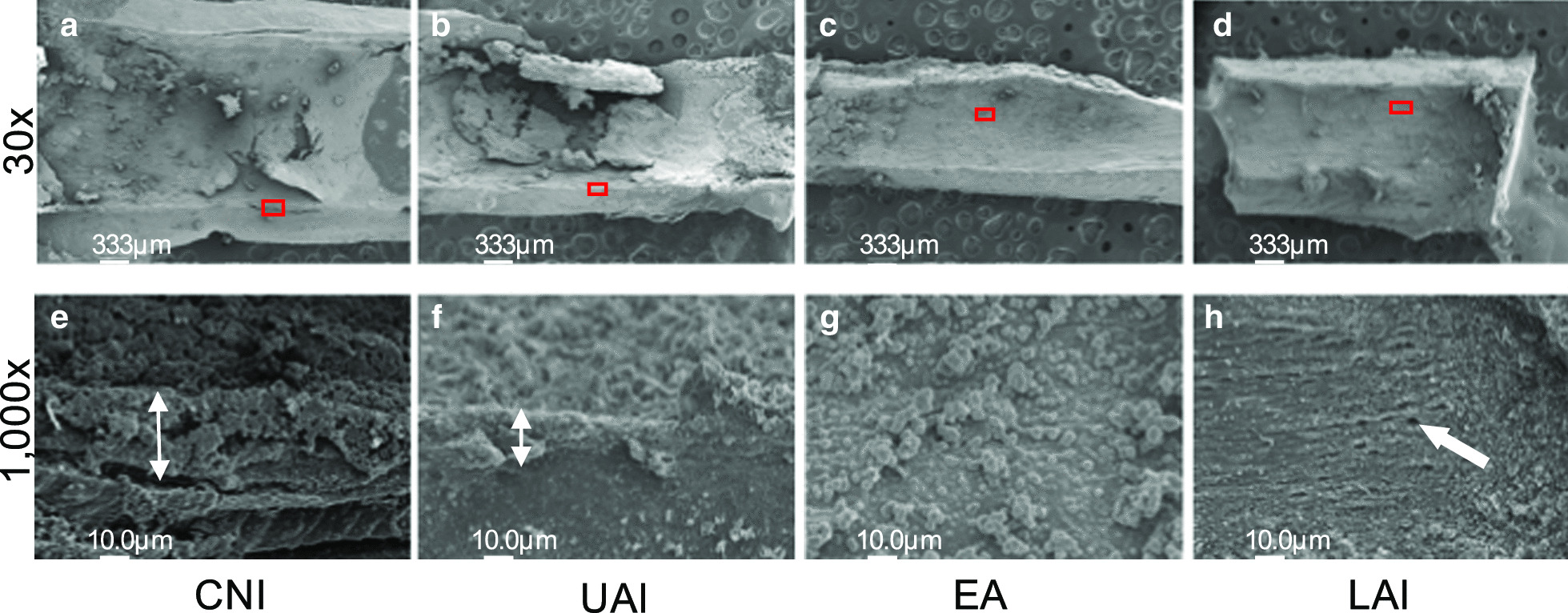
Morphological evaluations of each irrigation group. Effects of different root canal irrigation methods in the pig model including CNI (**a**, **e**), UAI (**b**, **f**), EA (**c**, **g**) and LAI (**d**, **h**) were investigated by SEM. **a**–**d** Images of the root canal wall at the apical one-third (30×). **e**–**h** Higher magnification views (1000×) of the red boxed areas are shown in the corresponding lower panel. **e**, **f** White double-headed arrows indicate the biofilm thickness. **h** White single-headed arrow denotes dentinal tubules. CNI: Conventional needle irrigation; UAI: Ultrasonically activated irrigation; EA: EndoActivator; LAI: Laser-activated irrigation

**Fig. 6:**
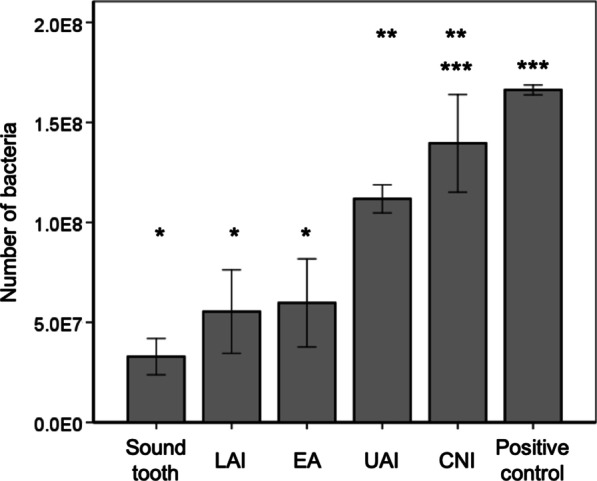
Quantitative analysis of each irrigation group. There were no significant differences between the positive control and CNI, CNI and UAI, or among sound tooth (negative control), LAI and EA groups (*Tukey Kramer* test*, p* < 0.05). The LAI and EA groups showed significant bacterial reduction compared with the UAI, CNI and positive control groups (*). CNI: Conventional needle irrigation; UAI: Ultrasonically activated irrigation; EA: EndoActivator; LAI: Laser-activated irrigation

We investigated the biofilm-cleaning ability of CNI, UAI, EA and LAI in the infected root canals in the pig model. The CNI and UAI groups still had debris attachment on the root canal wall (Fig. 5a, b), whereas EA and LAI resulted in lower debris compared with CNI and UAI (Fig. 5c, d). Higher magnification views revealed a multi-layered biofilm structure in the CNI and UAI groups (Fig. 5e, f), whereas the EA and LAI treatments showed fewer remnants of debris (Fig. 5g, h). The root canal surfaces that underwent LAI showed a slight opening of the dentinal tubules compared with the other groups (Fig. 5h). Quantitative PCR analysis further revealed that the number of bacteria in the infected root canal was most significantly reduced in the LAI (5.5 × 10^7^ cells) and EA (6.0 × 10^7^ cells) groups compared with the UAI (1.1 × 10^8^ cells) and CNI (1.4 × 10^8^ cells) groups (Fig. 6). In contrast, no significant differences were observed between the CNI and positive control groups (1.7 × 10^8^ cells), whereas the UAI group showed a lower bacterial number than the positive control group (Fig. 6). There were no significant differences observed between CNI and UAI groups, and among the sound tooth, LAI and EA groups (*Tukey Kramer* test,* p* < 0.05).

## Discussion

Many experimental models and approaches have been employed to date to evaluate the efficacy of root canal irrigation. Classically, radiopaque irrigants in vivo or dye solutions used in transparent root canal models in vitro have been utilized to monitor the penetration of these solutions [29, 40–46]. Artificially placed dentine debris using a split tooth is a simple method of determining the influence of irrigation and irrigant activation techniques by scoring the remaining debris [30, 47]. Organic tissues have also been used to evaluate chemical debridement and the efficacy of irrigant activation, and it has been revealed that ultrasonic activation enhances chemical debridement in simulated curved canals and accessory canals [31, 48]. Computational fluid dynamics (CFD) has also provided a further understanding of fluid flow mechanisms [10, 32, 49–51]. CFD studies have provided measurements of velocity magnitude, velocity vectors, and wall shear stresses with various needle designs and positioning. The effects of various irrigating solutions against endodontic biofilm have been assessed in previous reports, particularly from a chemical aspect, and optimal irrigant concentrations and temperatures have been described [26, 52–55]. Notably however, no in vivo study models had yet been developed to compare the efficacy of different irrigation protocols for clinical biofilm removal [56].

Pigs have been adopted as an experimental model in many biomedical fields due to some clear similarities with the human anatomy, and due to the obvious ethical considerations with regard to human subjects. Alveolar bone mineral contents, and the inflammation and destruction processes in periodontal tissues, are among the notable biological similarities between pigs and humans [35, 57]. In our current experimental pig model, we could successfully observe bone defects at the periapical area after exposing the root canal system to the oral environment. These defects developed as a consequence of inflammation, confirmed by an increased CRP level at 2 weeks after root canal exposure. As found in previous studies, intraradicular biofilms can arise through the opening of an access cavity for 2 weeks to enable contamination, and subsequent sealing for 4 weeks to produce an anaerobic environment [58, 59]. We observed typical biofilm thickness in the entire root canals in our pig model by SEM imagery in the positive control tooth. Importantly, we confirmed in our current experimental pig model that the most abundant and prevalent phyla within the intracanal biofilms were Firmicutes, Bacteroidetes and Fusobacteria*,* which predominate also in human samples [60]. Although, we did not use human samples in our current analyses, our results are comparable to those reported for human microbiota, based on previously described and robust experimental protocols [61, 62]. In our current study in the pig, we utilized the lower deciduous mandibular second premolars because this tooth length is similar to that in humans. Although the apical size of approximately 0.7–1.0 mm in diameter is wider, and the root dentin thickness is thinner, in the pig than in human permanent teeth, the same armamentarium used for root canal treatments in human clinical practice can be readily applied also in a pig model. The intraradicular biofilm pig model is therefore far more reflective of human conditions than those created using rodents or rabbits.

We focused in our present study on the chemical reduction of biofilm using NaOCl [61, 62] and agitating irrigation techniques. Hence, we did not utilize mechanical instrumentation nor EDTA irrigation. Although mechanical instrumentation is absolutely essential for the debridement of biofilm and to reduce the bacterial count from the root canal, it is obviously impossible to reach the whole root canal surface in this way [63]. To eradicate biofilm from these unreached areas, root canal irrigation and agitation are likely to be needed. A notable limitation of our current study however was that the tooth did not represent a curved canal. Future studies should consider comparing the efficacy of different techniques for the in vivo removal of an intraradicular biofilm from a curved root canal.

We used our pig model system to test the effectiveness of various established human irrigation protocols in removing biofilm from the root canal system. In terms of bacterial quantification however, it must be pointed out that the actual oral hygiene of the pig is quite poor. Thus, although calculous removal and tooth cleaning were performed in our pigs before extraction to reduce bacterial contamination, a sound tooth was used as a negative control for quantification analysis. Hence, although contamination by bacteria may occur during tooth extraction in the pig model system, our present results showed that all of the tooth samples with induced biofilm formation had a significantly higher number of bacteria than the sound tooth. In accordance with previous reports, the CNI method was found in our current analysis to be insufficient to clean the root canals due to its delivery limitations [61, 62]. Our findings indicated in fact that almost no biofilm was removed by CNI within five minutes. A large number of prior UAI studies have reported positive results in the removal of intracanal hard tissue debris and pulp tissue remnants due to the acoustic streaming generated by oscillating movements [12, 64, 65]. However, UAI was further found in a prior study to be less effective than chemo-mechanical preparation in a large canal [33], indicating that it is limited in terms of intraradicular biofilm removal from a wide root canal. Our current results in the pig model were consistent with this as we found no significant differences between the efficacy of CNI and UAI.

The sonic energy in the EA method has been found to generate a higher back-and-forth tip movement amplitude. The effectiveness of EA in cleaning an infected root canal and in smear layer removal is reported to be inferior or equal to that of UAI [66–68]. The main difference between EA and UAI is whether the tip of the device directly contacts the root canal surface or not. The range of the vibrating polymer tip of the EA is much wider than the range of motion of a UAI tip, and this increases the area where the tip makes physical contact with the root canal surface. Hence, our current results with EA in the pig model suggest that the generation of a mechanical force against the root canal wall is effective for eliminating firmly attached biofilms.

LAI induces significant fluid turbulent flow inside the root canal due to the generation and collapse of a vapor babble, improving the agitation of root canal irrigant. With the collapse of the bubble, secondary cavitation occurs, which could possess physical force allowing mechanical removal of a biofilm. A recent study has reported that the combination of extremely short laser pulses (50 µsec) and dual pulse irradiation generates shock waves that produce a larger physical force [69]. In our present study, we utilized a 300 µsec laser pulse duration, which is insufficient to generate shock waves, and inserted the fiber tip up to 3 mm short of the working length. Thus, the mechanical force leading to intraradicular biofilm removal was due to the collapse of a vapor bubble around the tip and secondary cavitation, which is consistent with a previous in vitro study [70]. Biofilm removal by non-contact physical force using a laser should be further investigated to improve its effectiveness.

## Conclusions

An experimental intraradicular biofilm model has been successfully established in the pig. Analyses using this model suggested that agitating root canal irrigants with sufficient physical reaction at the root canal wall could be used to disrupt and remove biofilm within a root canal. Our novel in vivo biofilm model in the pig will likely make important future contributions to improving the efficacy of root canal treatments.

## Data Availability

All the datasets used and analyzed during the current study are available from the corresponding author on reasonable request.

## Abbreviations

NaOCl: Sodium hypochlorite
CNI: Conventional needle irrigation
LAI: Laser-activated irrigation
CRP: C-reactive protein assay
UAI: Ultrasonically activated irrigation
EA: Endoactivator
OTU: Operational taxonomic unit
CFD: Computational fluid dynamics
